# Anti-oxidative trypanocidal drugs, myth or reality

**DOI:** 10.1186/2008-2231-21-21

**Published:** 2013-03-12

**Authors:** Soodabeh Saeidnia

**Affiliations:** 1 Medicinal Plants Research Center, Faculty of Pharmacy, Tehran University of Medical Sciences, Tehran 1417614411, Iran

## 

Sir,

“Blueberries best be eaten because they taste good, not because their consumption will lead to less cancer.” Prof. Jim Watson [1].

Recently, Professor Jim Watson, father of DNA double-helix, has described in his paper that antioxidant supplements may have caused more cancers than they have prevented. Alongside this controversial, several papers give rise on looking for anti-cancers among antioxidants [2-4]. In reality, mechanism of anti-cancer action is very important, where induction of free radicals and augmentation of Reactive Oxygen Species (ROS) are the main pharmacological modes of anti-neoplastic activity [5]. In spite of the fact that beneficial role of antioxidants in chemoprevention of cancer has been well-known, effectiveness of them in treatment of cancer is still in challenge.

Interestingly, there is a similar situation for another important disease, Chagas (American trypanosomiasis), a tropical parasitic illness caused by the flagellate protozoan *Trypanosoma cruzi* and affects eight to 10 million people residing in Latin American countries [6,7]. Nowadays natural compounds have been evaluated for discovery of new chemotherapeutics in treatment of Chagas disease. But, literature revealed that most of the researchers select the compounds without attention to their probable antioxidant activity, while therapeutic action of the remarkable trypanocidal compounds like elatol involves mitochondria as the primary target leading to increase of ROS generation through the electron transport chain, which consequently affects cell membrane and DNA integrity and finally death of parasite [8]. Although this is not the only mechanism of action for trypanocidal drugs, well-known natural phenolics such as gallic acid can act *via* pro-oxidant activity as well as enhancing DNA single-strand breaks to kill *Trypanosoma*[9]. Furthermore, anti-trypanosoma activity of komaroviquinone (a diterpenoid) was found to be attributed by generation of ROS catalyzed by *T. cruzi* old yellow enzyme [10]. It seems that potent natural antioxidants like hydroxycoumarins derivatives or flavonoids are not potentially strong trypanocidal agents [11].

The question arise from this concept is “why does a physician recommend consumption of antioxidant in treatment of trypanosomiasis?” The answer to this question may be hidden behind the differences in mechanism of actions for trypanocidal drugs and also the role of antioxidants in prevention of inflammation in this illness. To the best of our knowledge, consumption of antioxidant supplements is new perspective in therapy of Chagas disease because of the attenuation of oxidative stress associated to this disease [12]. Additionally, it has been reported that *Trypanosoma* infection decreases the levels of reduced glutathione (GSH) in the blood, liver, kidney, and also the plasma levels of vitamin C [13]. For this reason, they believe that supplementation of infected patient with vitamin C could prevent the depletion of endogenous antioxidants. Actually, another probable reason for this entrenched attitude is the ubiquitous application of antioxidant supplements. Therefore, we recommend the researchers to focus on possible mode of actions for trypanocidal candidates, especially when the nominated compounds are proved to possess antioxidant ability.

## Competing interest

The author declared that there is no competing interest.
